# A novel specific duplex real-time RT-PCR method for absolute quantitation of *Grapevine Pinot gris virus* in plant material and single mites

**DOI:** 10.1371/journal.pone.0197237

**Published:** 2018-05-15

**Authors:** Félix Morán, Antonio Olmos, Leonidas Lotos, Lukáš Predajňa, Nikolaos Katis, Miroslav Glasa, Varvara Maliogka, Ana B. Ruiz-García

**Affiliations:** 1 Centro de Protección Vegetal y Biotecnología, Instituto Valenciano de Investigaciones Agrarias (IVIA), Moncada, Valencia, Spain; 2 Aristotle University of Thessaloniki, Faculty of Agriculture, Forestry and Natural Environment, School of Agriculture, Plant Pathology Laboratory, Thessaloniki, Greece; 3 Institute of Virology, Biomedical Research Centre, Slovak Academy of Sciences, Dúbravská cesta 9, Bratislava, Slovakia; Universita del Salento, ITALY

## Abstract

*Grapevine Pinot gris virus* (GPGV) is a widely distributed grapevine pathogen that has been associated to the grapevine leaf mottling and deformation disease. With the aim of better understanding the disease epidemiology and providing efficient control strategies a specific and quantitative duplex TaqMan real-time RT-PCR assay has been developed. This method has allowed reliable quantitation of the GPGV titer ranging from 30 up to 3 x 10^8^ transcript copies, with a detection limit of 70 viral copies in plant material. The assay targets a grapevine internal control that reduces the occurrence of false negative results, thus increasing the diagnostic sensitivity of the technique. Viral isolates both associated and non-associated to symptoms from Greece, Slovakia and Spain have been successfully detected. The method has also been applied to the absolute quantitation of GPGV in its putative transmission vector *Colomerus vitis*. Moreover, the viral titer present in single mites has been determined. In addition, in the current study a new polymorphism in the GPGV genome responsible for a shorter movement protein has been found. A phylogenetic study based on this genomic region has shown a high variability among Spanish isolates and points to a different evolutionary origin of this new polymorphism. The methodology here developed opens new possibilities for basic and epidemiological studies as well as for the establishment of efficient control strategies.

## Introduction

*Grapevine Pinot gris virus* (GPGV) is a member of the genus *Trichovirus*, family *Betaflexiviridae*. GPGV was described for the first time in Italy as a new trichovirus associated to the grapevine leaf mottling and deformation disease that causes a variety of symptoms, such as chlorotic mottling, puckering and deformations of the leaves, reduced yields and low quality of the berries [1]. GPGV genome consists of one molecule of positive single stranded RNA, ranging from 7223 to 7275 nucleotides excluding the 3’ polyA tail. The genome contains three overlapping open reading frames (ORFs) and two untranslated regions (UTRs) located at both the 5’ and the 3’ ends. ORF1 encodes a methyltransferase, a helicase and the RNA dependent RNA polymerase (RdRp), ORF2 encodes the movement protein (MP) and ORF3 encodes the coat protein (CP). Several studies have reported the existence of high variability in GPGV genomes [2–4]. Among this genomic diversity, a polymorphism involving the MP stop codon that produces a six amino acids shorter protein has been described [3]. Although this polymorphism has been related to the symptoms observed in GPGV infected grapevines, a association between this genetic variant and the manifestation of symptoms remains unclear. Phylogenetic studies based on the GPGV MP/CP genomic region have allowed to cluster GPGV isolates in different clades, being some of them generally associated to the presence or absence of symptoms. [3, 4]. In the field, the GPGV-infected grapevines are often simultaneously affected by other viruses, making difficult to attribute a specific etiology to the GPGV infection. In addition, other factors such as viral titer have been reported to play an important role in the development of the disease [4].

GPGV seems to have a high worldwide distribution, as it has been reported infecting grapevine in Italy, Greece, Czech Republic, Slovakia, Slovenia, France, Germany, Portugal, South Korea, China, Canada, USA, Spain, Turkey, Brazil, Croatia, Romania, Ukraine and Australia [5–9]. GPGV has also been shown to infect and induce symptoms in the herbaceous hosts, *Silene latifolia* subsp. *alba* (Mill.) and *Chenopodium album* L. which might represent natural reservoirs of the virus [10]. A potential transmission vector for GPGV has been found, the eriophyid mite *Colomerus vitis*, which has been described to transmit the virus to healthy grapevines [11].

The broad distribution of this pathogen, the existence of potential transmission vectors and reservoirs and the effect of the disease in grape production [1,4] highlight the need for the performance of future studies that give insights into the disease epidemiology and provide efficient control strategies. The main control strategies of plant viral diseases lay on early detection, eradication and use of genetically resistant or tolerant cultivars. In this scenario, the use of specific and reliable detection methods becomes a key factor in the management and control of the diseases. Several detection methods have been developed for the diagnosis of GPGV, based on biological indexing [3] and conventional RT-PCR [2, 12]. Recently, a real-time RT-PCR detection method based on SYBR Green chemistry that allows a relative estimation of the virus titer has been developed by Bertazzon et al. [4]. Quantitative real-time PCR methods have been shown to be powerful tools for viral epidemiological studies and viral detection [13–17]. The aim of this work was the development of a specific real-time RT-PCR detection method able to perform an absolute quantitation of GPGV. This method would represent a powerful tool for GPGV detection and could be applied to future epidemiological studies, allowing the quantitation of the number of viral targets present in plant samples and transmission vectors.

## Materials and methods

### Virus isolates and plant material

Three GPGV isolates from Slovakia (SK30, SK45 and SK704), three isolates from Greece (Tra7, Tra9 and Amb1) and two Spanish isolates (RQ30 and RQ25) were used as positive controls in this study [2,5]. Plant material from three grapevine cultivars sanitized by tip culture was used as negative control. In addition, 178 grapevine samples from a random survey on a Spanish grapevine growing area (D.O. Utiel-Requena) were analyzed. The geographic coordinates of the two fields of study obtained by Google Maps were 39.47° N 1.18° W (San Antonio) and 39.63° N 1.27° W (Las Cuevas). The survey was conducted on private land, under the permission of the owners.

### RNA isolation

Total RNA from leaf tissue was extracted using the Plant/Fungi Total RNA Purification Kit (Norgen Biotek Corporation, Thorold, ON, Canada) following the manufacturer instructions. RNA was quantified with a NanoDropND-100 spectrophotometer (NanoDrop Technologies) to determine the RNA concentrations and stored at -80°C until subsequent analysis.

### Sequence alignment and primers and probe design

Sequence alignment of 96 GPGV sequences available in the databases (https://www.ncbi.nlm.nih.gov, accessed on September 2017) was performed using Mega 6 software [18]. Primers were designed on a highly conserved region of the GPGV genome corresponding to the MP/CP genes, GPGV-RT-F (5’-GTCTAAATCTGGCTGTGCTGAAAATAGTGC-3’) and GPGV-RT-R (5’-CCTGAGGTCCCTTCAACTGC-3’), yielding an amplicon of 228 bp. A TaqMan ZNA probe, GPGV-genprobe (5’-6-FAM-AGATCAACAGTCAGGAGAGAGCTGATCGC-ZNA4-BHQ1-3’) was designed inside the amplified region. The *Vitis vinifera* housekeeping phosphoenolpyruvate carboxylase gene (*PEP*) was used as an internal control for the real-time RT-PCR reaction [19]. Primers PEP-F1 (5’- GCCTCCTCCTCCAGATTGCT-3’) and PEP-F2 (5’- AGGCTTGCTTGATTCCATTATCTCTTTCG-3’) were used to amplify a fragment of 196 bp. A TaqMan ZNA probe was designed inside this fragment, PEP-probe (5’-Cy5- CGACCCATACTTGAAACAGAGACTCCGGC-ZNA-BHQ2-3’).

### Generation of real-time RT-PCR standard curves

A specific nucleotide sequence of the GPGV genome (438bp) containing the sequence targeted by the real-time RT-PCR assay was amplified by RT-PCR using the primers GPGV-6474F (5’-TTCTGGTGATCCAATGGTAAAGA-3’) and GPGV-6912R (5’-ATTGCAAAGGCCGCACACACTTG-3’). The amplicon was purified using the mi-PCR Purification Kit (metabion international AG, Martinsried, Germany), inserted in the vector pGEM-T Easy (Promega Corporation, Madison, USA) and cloned into *E*. *coli* HB-101. Transformants were selected by ampicillin resistance and the presence and orientation of the fragment evaluated by sequencing. The plasmid was linearized by restriction with *Sal* I and used as a target in an *in vitro* transcription assay using T7 RNA polymerase (Takara Bio Inc., Kusatsu, Japan) followed by DNA digestion by RQ1 RNase-free DNase (Promega Corporation, Madison, USA) at 37°C for 30’. RNA was purified using a Plant/Fungi Total RNA Purification Kit spin column (Norgen Biotek Corporation, Thorold, ON, Canada) and quantified as mentioned above. The quantitation of the RNA transcripts in picomoles was performed considering the average molecular weight of a ribonucleotide (340 Da) and the number of bases of the transcript (*N*_b_). The following mathematical formula was applied: pmol of ssRNA = (μg of ssRNA × 10^6^) / (340 × *N*_b_). Avogadro constant [20] was used to estimate the number of transcripts (6.023 × 10^23^ molecules/mol). Three replicates of ten-fold serial dilutions of the transcripts from 3 × 10^8^ to 3 × 10^1^ were prepared and used to generate the standard curve. The slope of the calibration curve was used to calculate the amplification efficiency, according to the mathematical formula: amplification efficiency = [10 ^(−1/slope)^]−1 [21].

### TaqMan quantitative real-time RT-PCR

TaqMan assays for quantitative real-time RT-PCR were carried out in a LightCycler 480, using AgPath-ID One-step RT-PCR kit (Ambion Inc., Austin, TX, USA) and 3 μl containing 60 ng of RNA as template. The reaction mixture contained either 150 nM of the probe GPGV-genprobe and 0.9 μM of each of the primers GPGV-RT-F and GPGV-RT-R (in a singleplex reaction) or 150 nM of the probe GPGV-genprobe, 50 nM of the probe PEP-probe, 0.9 μM of each of the primers GPGV-RT-F and GPGV-RT-R and 0.1μM of each of the primers PEP-F1 and PEP-R1 (in a duplex reaction with the plant internal control). RT-PCR protocol consisted of one step of 45°C for 10 min and 95°C for 10 min followed by 45 cycles of amplification (95°C for 15 s and 60°C for 1 min). Data acquisition and analysis were performed using the LightCycler 1.5 software included in the equipment. The default threshold set by the machine was slightly adjusted above the noise to the linear part of the growth curve at its narrowest point, according to the manufacturer.

### Sensitivity comparison of the TaqMan quantitative real-time RT-PCR with a SYBR Green real-time qPCR

The sensitivity of the TaqMan real-time method developed in this study was compared to a SYBR Green real-time RT-PCR protocol targeting two GPGV genomic regions, the RdRp and the CP [4]. Three replicates of ten-fold serial dilutions of GPGV infected plant extracts with healthy plant extracts were tested by both methods.

### Detection of other grapevine viruses

All the grapevine samples that tested positive for GPGV were analyzed for the presence of the following grapevine viruses: *Grapevine leafroll-associated virus 1* (GLRaV-1), *Grapevine leafroll-associated virus 2* (GLRaV-2), *Grapevine leafroll-associated virus 3* (GLRaV-3), *Grapevine fleck virus* (GFkV), *Grapevine fanlef virus* (GFLV) and *Arabis mosaic virus* (ArMV). Detection of these viruses was performed by real time RT-PCR, according to published protocols. GLRaV-1, GLRaV-3, GFkV, GFLV and ArMV presence was analyzed according to Bertolini et al. [22]. Detection of GLRaV-2 was carried out according to Osman et al. [23].

### MP polymorphism of GPGV Spanish isolates

Conventional RT-PCR was used to amplify the 228 bp fragment of the MP/CP genes targeted by the real-time assay in 20 GPGV positive samples using AgPath-ID One-step RT-PCR kit (Ambion Inc., Austin, TX, USA). The reaction mixture contained 0.9 μM of each of the primers GPGV-RT-F and GPGV-RT-R and 3 μl of RNA. RT-PCR protocol consisted of one step of 45°C for 30 min and 95 ºC for 10 min followed by 45 cycles of amplification (95°C for 30 s, 50°C for 30 s and 60°C for 1 min). Sanger sequences were aligned using Mega 6 software [18].

### Phylogenetic analysis of GPGV Spanish isolates

A 548 bp fragment of the GPGV MP/CP gene was amplified from 12 GPGV Spanish isolates by conventional RT-PCR using the primers Det-F and Det-R [3,4] under the conditions described above. Sanger sequences were aligned using Mega 6 software [18]. Maximum likelihood tree was obtained using the best nucleotide substitution model (Tamura 3). The sequences included in the phylogenetic analysis were RQ25, RQ30, RQ99, RQ106, RQ108, RQ111, RQ113, RQ115, RQ116, RQ117, RQ125 and RQ136 (GenBank accession numbers MH019203, MH019204, MH019205, MH019206, MH019207, MH019208, MH019209, MH019210, MH019211, MH019212, MH019213 and MH019214, respectively). The following reference sequences were included in the alignment: MOLA 14, MOLA 6, ALA-P4, ORM-G40, SUS-G49, PIA-G44, SK30 and SK01 (GenBank accession numbers LN606705, LN606703, LN606739, KU845367, KU845372, KU845348, 543887400 and 543887404, respectively).

### Quantitative GPGV detection on *Colomerus vitis*

Leaves with erinea produced by *C*. *vitis* were collected from GPGV-free grapevines and mites were transferred by contact to GPGV infected leaves. After five days of contact, mites were collected from the infected leaves and deposited on eppendorf tubes. Total RNA was extracted using a Plant/Fungi Total RNA Purification Kit (Norgen Biotek Corporation, Thorold, ON, Canada) with slight modifications. Briefly, 100 μl of lysis buffer was added to tubes containing 1, 5, 10 or 15 mites. All the assays were performed in duplicates and repeated five times. The suspension was vortexed for 2’ in the presence of 212–300 μm glass beads (Sigma-Aldrich, Misuri, USA). After 5’ centrifugation at 10000 x g the clarified supernatant was transferred to a fresh tube and the extraction procedure was completed as indicated by the manufacturer. The presence and quantitation of GPGV in *C*. *vitis* samples was performed as described above, except that a singleplex assay, using only GPGV primers and probe, was performed.

## Results

### Detection of GPGV by real-time RT-PCR

The ability of the real-time RT-PCR method designed in this work to detect GPGV was tested using the positive control from different geographical locations. Two independent replicates of three isolates from Greece (Tra7, Tra9 and Amb1), three isolates from Slovakia (SK30, SK45 and SK704) and two Spanish isolates (RQ30 and RQ25) were analyzed. For all GPGV infected samples a positive detection of the viral sequence was achieved, with a cycle threshold (Ct) values ranging from 24.2 to 30.7. No signal was detected for the three sanitized grapevine cultivars used as negative controls.

### Validation of a plant internal control

**Fig 1:**
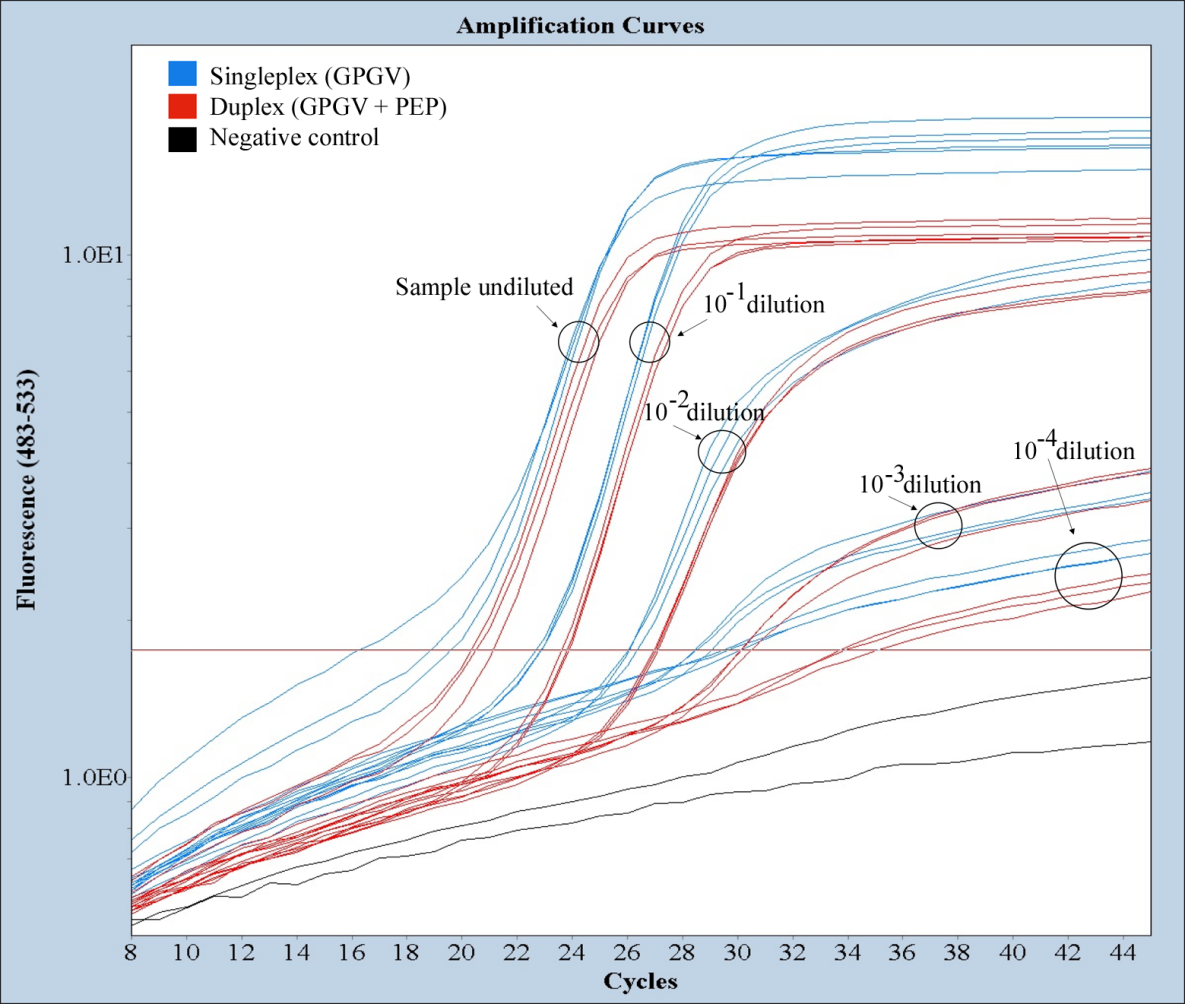
Validation of the *V*. *vinifera PEP* internal control in the duplex real-time RT-PCR GPGV assay. Three replicates of a GPGV infected plant extract were ten-fold serially diluted with healthy plant extract. Amplification plots by GPGV singleplex (blue) and GPGV-*PEP* duplex (red) real time RT-PCR assays are shown. Two negative controls (black) are included.

In order to decrease the number of putative false negative results, namely positive samples not detected by the method, an internal control of the grapevine *PEP* gene (phosphoenolpyruvate carboxylase) was included in the assay as a duplex RT-PCR reaction. Thus, a negative GPGV detection result is taken into account only if the internal control gives a positive signal. The possible effect of a duplex RT-PCR reaction in GPGV detection technical sensitivity was assessed by the comparison of the signal obtained in a singleplex assay (using only GPGV primers and probe) and the signal obtained in a duplex assay (including the plant internal control primers and probe) in several ten-fold serial dilutions of GPGV infected plant extracts with healthy plant extracts (Fig 1). Three replicates of each dilution were analyzed. The averages of the Ct values obtained and their standard errors were 21.75±0.22 (undiluted), 24.60±0.11 (10^−1^ dilution), 27.99±0.13 (10^−2^ dilution), 32.22±1.44 (10^−3^ dilution) and 37.68±0.43 (10^−4^ dilution), for the singleplex assay, and 22.54±0.16 (undiluted), 25.112±0.06 (10^−1^ dilution), 28.80±0.04 (10^−2^ dilution), 31.24±1.31 (10^−3^ dilution) and 36.79±1.03 (10^−4^ dilution), for the duplex assay. Student’s t-test was performed to compare the Ct values obtained for the three replicates in both conditions. No significance differences at 95% confidence were found. Therefore, GPGV was detected in both assays with similar Cts, which means that the use of an internal control does not compromise the ability of the method to successfully detect the virus.

### Absolute quantitation and sensitivity of the detection method

**Fig 2:**
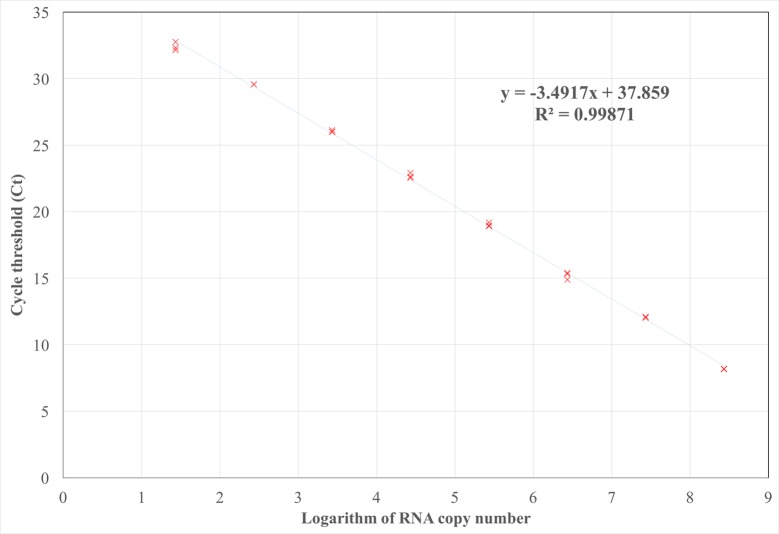
Absolute GPGV quantitation standard curve. Cycle threshold (Ct) values obtained for three replicates of ten-fold serial dilutions of GPGV control transcripts are plotted. The mathematical equation of the standard curve used to quantify the real-time RT-PCR assays and the coefficient of correlation (R^2^) are indicated.

Absolute quantitation of GPGV was performed using known quantities of *in vitro* synthesized GPGV transcripts of a region of the MP/CP genes. Three replicates of ten-fold serial dilutions of the transcripts allowed to establish a quantitation range from 3 × 10^8^ to 30 transcript copies (Fig 2). The slope of the standard curve (-3.49) was used to calculate an amplification efficiency of 93.43% with a coefficient of correlation (R^2^) of 0.99. The detection limit of the method was slightly higher when plant material was analyzed, being 70 the minimum amount of viral copies detected (data not shown).

The sensitivity of the GPGV quantitative real-time RT-PCR method developed was compared to that of the real-time qPCR method based on SYBR Green chemistry previously reported [4]. Several ten-fold serial dilutions of GPGV infected plant extracts with healthy plant extracts were tested by both methods (Table 1). The real-time RT-PCR developed in this study was able to detect the virus up to two ten-fold dilutions further than the previously reported method, showing thus a higher sensitivity for GPGV detection.

**Table 1:** Sensitivity comparison between TaqMan and SYBR Green GPGV real-time RT-PCR detection methods.

			Serial dilutions^a^						
qPCR method	Targeted region	Undiluted extract	10^−1^	10^−2^	10^−3^	10^−4^	10^−5^	10^−6^	10^−7^
TaqMan (this study)	MP/CP	+	+	+	+	+	-	-	-
SYBR Green (Bertazzon et al. [4])	RdRp	+	+	+	-	-	-	-	-
	CP	+	+	+	-	-	-	-	-

^a^ Ten-fold serial dilutions of GPGV infected plant material with healthy plant extracts

### Use of the duplex quantitative real-time RT-PCR for GPGV diagnosis

A random survey was conducted to evaluate the incidence of GPGV in the D.O. Utiel-Requena, one of grapevine growing areas where GPGV was detected for the first time in Spain [5]. GPGV was detected in 22 from a total of 178 samples analyzed by quantitative real-time RT-PCR, corresponding to plants from three different grapevine local varieties (Bobal, Macabeo and Tintorera). In 5 negative samples, no amplification of the internal plant control was obtained. Consequently, these putative false negative results were discarded. Thus, the estimated prevalence of the virus in these vineyards was 12.7%. The Ct values observed ranged from 18.5 to 33.8 which corresponded to a titer between 1.1 x 10^8^ to 1.6 x 10^3^ viral copies detected (Table 2). Evaluation of symptoms in these samples revealed the presence of GPGV in both plants showing chlorotic mottling and asymptomatic grapevines.

**Table 2:** Real-time RT-PCR cycle threshold (Ct) values for GPGV and *PEP* internal control, absolute quantitation, symptomatology and presence of other grapevine viruses in GPGV positive samples collected from D.O. Utiel-Requena.

Sample	Variety	Ct Values (GPGV^a^)	Viral titer	Ct Values (*PEP)*	Symptoms^b^	Other viruses^c^
RQ25	Bobal	25.0	1.7 x 10^5^	20.2	CM, LD	GFLV, GLRaV-3
RQ30	Bobal	23.4	3.9x 10^5^	21.6	CM, LD	GFkV, GFLV, GLRaV-3
RQ99	Macabeo	21.2	1.3 x10^6^	21.3	CM	GFLV, GFkV, GLRaV-2
RQ106	Macabeo	23.1	4.5 x10^5^	20.6	SL	GLRaV-2
RQ108	Macabeo	23.2	4.4 x 10^5^	21.5	CM	GFLV
RQ109	Macabeo	23.2	4.4 x 10^5^	18.7	CM	GFLV
RQ110	Macabeo	23.2	4.4 x 10^5^	19.7	CM	GFLV
RQ111	Tintorera	22.9	5.2 x 10^5^	21.7	CM	GFLV
RQ113	Tintorera	20.5	1.8 x 10^6^	21.6	CM	GFLV
RQ115	Tintorera	24.7	2.0 x 10^5^	21.9	CM	GFLV
RQ116	Bobal	26.6	7.5 x 10^4^	22.0	CM	-
RQ117	Bobal	18.5	1.1 x 10^8^	21.9	CM	-
RQ118	Bobal	27.5	4.6 x 10^4^	22.8	CM	-
RQ121	Tintorera	32.3	3.6 x 10^3^	22.7	SL	GLRaV-3, GFkV
RQ124	Tintorera	30.9	7.8 x 10^3^	23.5	SL	GFLV
RQ125	Tintorera	23.2	4.3 x 10^5^	22.7	SL	GFLV, GLRaV-2
RQ136	Bobal	24.2	2.6 x 10^5^	20.3	CM	GFLV
RQ140	Bobal	31.5	5.7 x 10^3^	20.7	CM	GFLV
RQ142	Bobal	32.6	3.2 x 10^3^	20.5	SL	GLRaV-2, GLRaV-3
RQ143	Bobal	33.8	1.6 x 10^3^	21.0	CM	GLRaV-2, GLRaV-3
RQ145	Macabeo	32.0	4.2 x 10^3^	20.5	CM	GFLV
RQ150	Macabeo	33.2	2.3 x 10^3^	20.7	CM	GFLV
RQ151	Macabeo	33.0	2.6 x 10^3^	20.8	CM	GFLV
RQ160	Tintorera	32.7	3.0 x 10^3^	24.0	CM	GFLV

^a^ *Grapevine Pinot gris virus* (GPGV)

^b^*Chlorotic mottling* (CM); *Leaf deformation* (LD); *Symptomless* (SL).

^c^*Grapevine fanleaf virus* (GFLV); *Grapevine leafroll-associated virus 2* (GLRaV-2); *Grapevine leafroll-associated virus 3* (GLRaV-3); *Grapevine fleck virus* (GFkV).

### Presence of other grapevine viruses

The possible presence of other grapevine viruses known to infect and produce symptoms in grapevine was evaluated. The results of this study showed that most of the GPGV infected samples detected (19 out of 22) were co-infected with one or more grapevine viruses tested (Table 2). GFLV was the most common co-infecting virus, being present in 15 of the 22 samples. GFkV, GLRaV-2 and GLRaV-3 were also detected in some samples. The presence of mixed infections was detected in both symptomatic and symptomless plants.

### New polymorphism on the GPGV MP/CP genes of Spanish isolates

**Fig 3:**
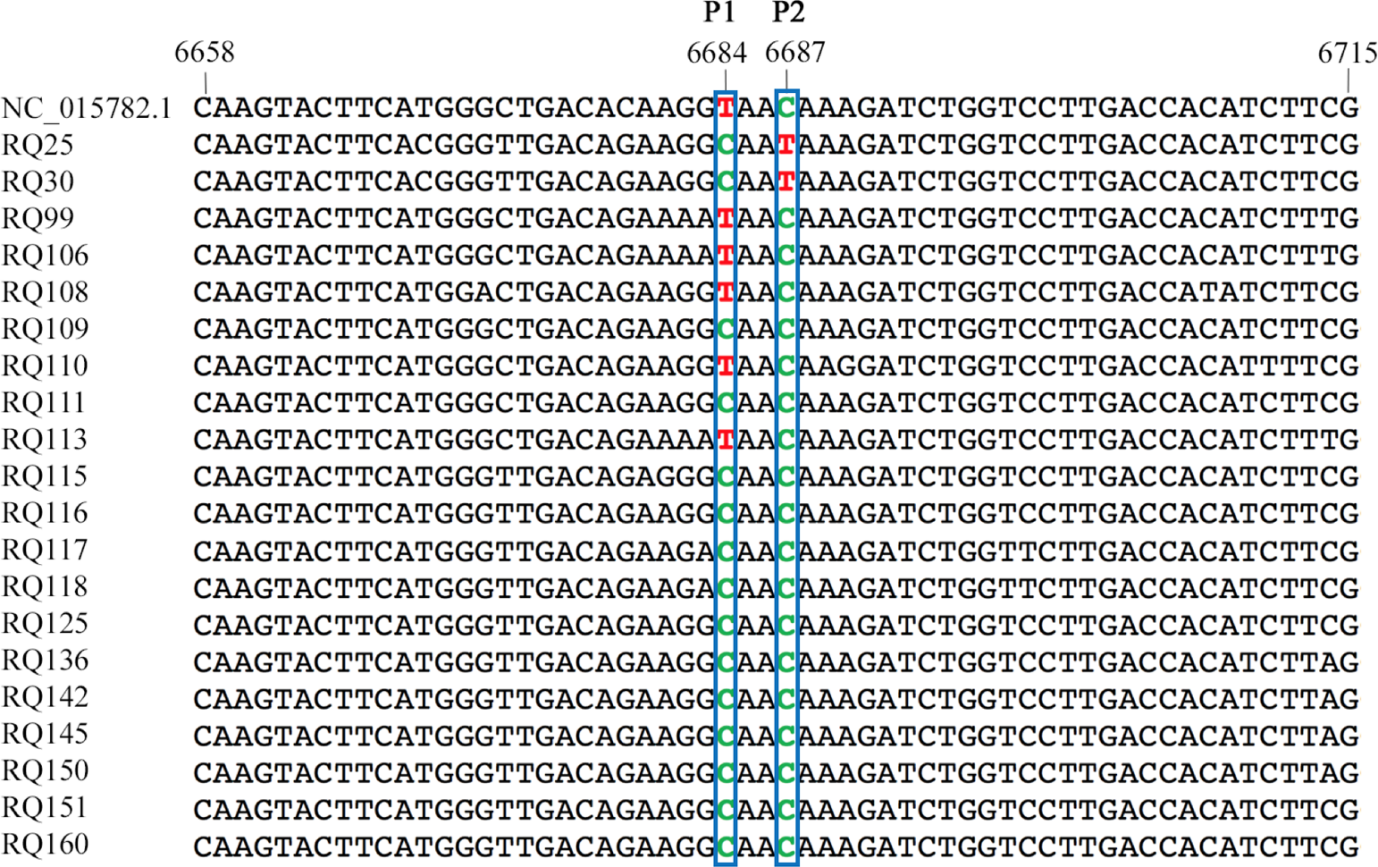
Sequence alignment of the MP/CP region targeted by the real-time RT-PCR assay of 20 GPGV Spanish isolates using GPGV IT isolate (NC_015782.1). Two polymorphisms producing a shorter MP: P1 (previously reported by Saldarelli et al. [3]) at position 6,684 and P2 (new polymorphism identified in this work) at position 6,687 are indicated.

The 228 bp MP/CP fragment targeted by the real-time RT-PCR was successfully amplified by conventional RT-PCR in 18 of the GPGV infected plants detected and in two Spanish positive controls (RQ30 and RQ25). Sanger sequencing of these amplicons showed the presence of a new polymorphism in the MP/CP gene, not previously reported (Fig 3). This polymorphism, detected in the Spanish isolates RQ30 and RQ25, involves the presence of a translational stop signal one codon downstream from the polymorphism reported by Saldarelli et al. [3]. The presence of this single nucleotide polymorphism would determine the synthesis of a five amino acids shorter MP. Although both RQ30 and RQ25 show GPGV related symptoms, such as chlorotic mottling and leaf deformation, this polymorphism is not present in other symptomatic plants. In 4 of the samples analyzed (RQ99, RQ106, RQ108 and RQ113) the polymorphism previously reported in the literature was found in both symptomatic and symptomless plants.

### Phylogenetic study of GPGV Spanish isolates

**Fig 4:**
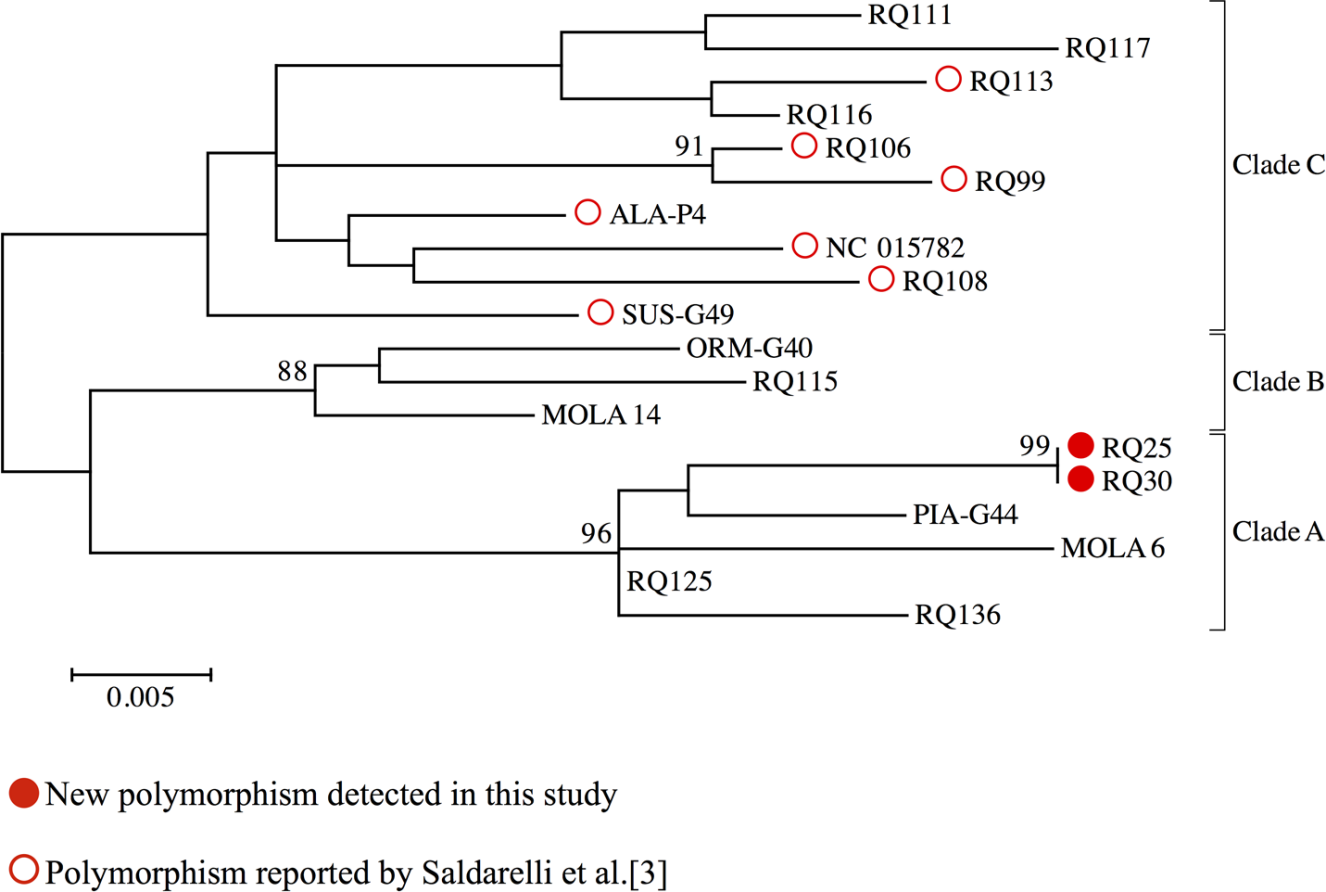
Phylogenetic analysis of Spanish GPGV isolates by Maximum likelihood method. Trees were generated under the Tamura 3 model of nucleotide substitution. Branches supported by a minimum of 75% of 2,000 bootstrap replicates.

In order to study the phylogenetic significance of the new polymorphism found in the GPGV MP/CP region, a phylogenetic study based on a 548 pb fragment of the MP/CP gene was conducted. A phylogenetic tree, including 12 Spanish isolates representing different polymorphisms and 8 reference sequences representative of the three clades (A, B and C) identified by Saldarelli et al. [3] and Bertazzon et al. [4], was constructed (Fig 4). Spanish isolates did not cluster in any particular clade but were distributed along all the three clades. Interestingly, the two isolates (RQ30 and RQ25) showing the new MP/CP polymorphism did not group in the same clade where isolates with the polymorphism previously reported by Saldarelli et al. [3] are located. Isolates containing the new polymorphism seems to be phylogenetically classified to a different clade (identified as clade A by Saldarelli et al. [3] and Bertazzon et al. [4]).

### Determination of the GPGV titer in the putative transmission vector *Colomerus vitis*

**Fig 5:**
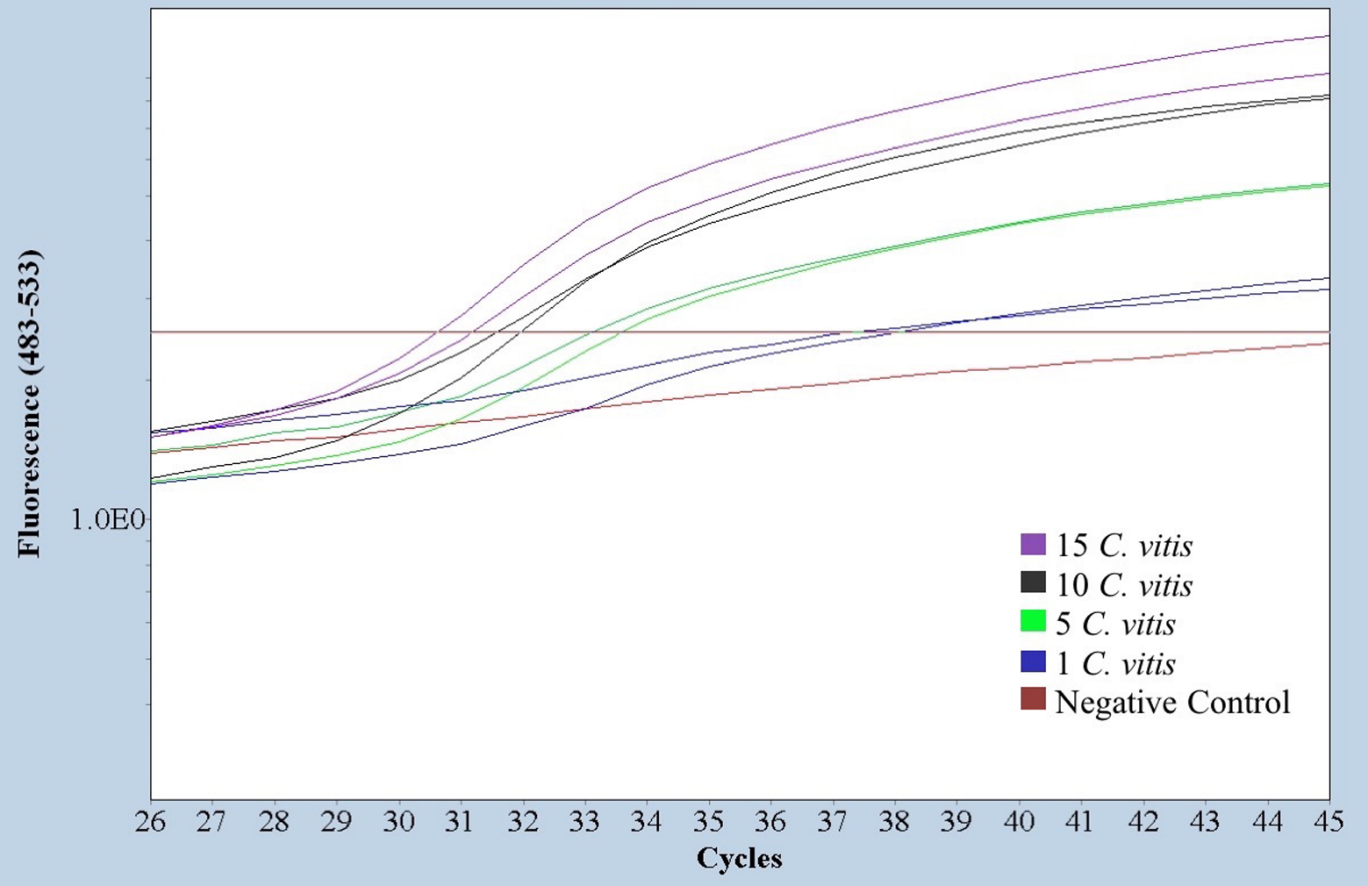
Absolute quantitation of GPGV in the putative transmission vector *C*. *vitis*. Amplification plots obtained for pools of 15 (purple), 10 (black) and 5 (green) and individual mites (blue) are shown. A negative control of mites fed on GPGV free leaves (brown) is included.

The real-time RT-PCR method developed in this study was applied for the quantitative detection of GPGV in the putative transmission vector *C*. *vitis*. Pools of 15, 10, 5 and single mites fed on GPGV infected fresh leaves were analyzed after an acquisition period of five days. GPGV was successfully detected in all samples, including single mites (Fig 5). *C*. *vitis* fed on healthy grapevine leaves were used as negative controls. The average viral titer in a single *C*. *vitis* was 306 (standard error ±6) copies of GPGV under our experimental conditions. The average viral titer calculated in the pools gave a quantification of 161±15 viral copies per mite.

## Discussion

Quantitative real-time PCR methods have been shown to be powerful tools for viral epidemiological studies and viral detection. In this study, we report the development of a specific real-time RT-PCR detection method able to perform an absolute quantitation of GPGV in both plant material and transmission vectors, which represents a useful and reliable tool in the diagnosis and study of this plant pathogen.

The method here described has been designed as a duplex RT-PCR reaction that includes an internal plant control, the *V*. *vinifera* phosphoenolpyruvate carboxylase gene (*PEP*) reported by Borges et al. [19] as a useful housekeeping gene for transcriptional studies performed by real-time PCR. The results presented in this work show the absence of a negative effect of the duplex reaction on the GPGV technical sensitivity of the method, as the signal obtained by the duplex reaction was similar to that obtained in a singleplex assay. In addition, the sensitivity in the detection of GPGV of the TaqMan method developed here is higher that the sensitivity of a real-time qPCR method based on SYBR Green chemistry previously reported [4]. Moreover, the diagnostic sensitivity, that is the ability of a technique to detect the true positives among all the infected plants: sensitivity = true positives / (true positives + false negatives) [24] is increased in our method, as the use of a grapevine internal control in the real-time RT-PCR reduces the number of false negative results. In fact, some of the plants analyzed in this study that tested negative for GPGV did not provide a positive detection on the grapevine *PEP* gene, allowing the identification of these samples as putative false negative results. To our knowledge this is the first GPGV detection method that includes such plant internal control.

This method also allows the absolute quantitation of GPGV titer not only in plant material but also in transmission vectors. In fact, we have been able to determine the viral titer present in a single *C*. *vitis* mite. The average number of copies detected in single mites was 306±6. Interestingly, the viral titer calculated in mite pools gave an average number of copies per mite (161±15) that lied in the same order of magnitude, suggesting a homogeneous distribution of the amount of viral particles present in this putative GPGV transmission vector.

The real-time RT-PCR described in this work has been used to study the incidence of GPGV in the grapevine growing area D.O. Utiel-Requena. The estimated prevalence of the virus found in these vineyards was 12.7%. Only some of the infected plants, 17 of 22 showed GPGV related symptoms, as chlorotic mottling, being asymptomatic the rest of the isolates detected. The presence of other grapevine viruses (such as GFLV, GFkV, GRLaV-2 and GLRaV-3) has been detected in many of the GPGV infected plants. However, no clear association was found between the symptomatology observed and the presence of these mixed infections, as some of the asymptomatic samples also showed GPGV and other viruses co-infections. Other factors such as viral titer have been reported to be involved in the development of symptoms [4] highlighting the importance of the performance of an accurate and absolute quantitation of GPGV. In this study, no clear association between the symptoms and GPGV titer has been observed, however the reduced number of isolates analyzed due to the low incidence of GPGV in the grapevine growing area surveyed could explain this finding. A more exhaustive survey on different GPGV affected Spanish vineyards could help to address this issue.

The new method targets the MP/CP gene, an interesting region of GPGV genome where other authors have reported the existence of a polymorphism responsible for a six amino acids shorter MP [2, 3]. The amplification and sequencing of this genomic region of Spanish isolates has allowed us to identify a new polymorphism in this gene. This polymorphism is located one codon downstream the previously reported one and has a similar effect on GPGV MP, making the protein five amino acids shorter in this case. The phylogenetic study of these genome variants showed that the new polymorphism clusters in a different clade than the polymorphism previously reported in the literature. This phylogenetic divergence suggests that the existence of these two different polymorphisms that result in a similar shortening of the GPGV MP are independent evolutionary events which biological significance remains unclear. The finding of a new phylogenetically unrelated polymorphism gives new insights in the study of GPGV genomic variability. The symptomatology observed in this study in the Spanish local varieties analyzed did not seem to be correlated to the MP/CP polymorphism reported by Saldarelli et al. [3] nor with the new polymorphism detected in this work. These findings raise an important issue about GPGV symptomatology that needs to be addressed taking into account multiple factors that may interact in the development of the grapevine leaf mottling and deformation disease. Further characterization of GPGV Spanish isolates and their phylogenetic relationships, the study of the susceptibility of the local grapevine varieties and the use of cDNA infectious clones will help to understand the biological significance of this emergent pathogen.

In conclusion, we report the development of a novel specific and quantitative GPGV detection method based on TaqMan real-time RT-PCR. This method, that allows the absolute quantitation of GPGV, improves the diagnostic sensitivity of GPGV detection by the false negative results reduction provided by the use of a plant internal control. Accurate estimation of the number of virions might be essential in understanding the development of the grapevine leaf mottling and deformation disease. Moreover, the method here described allows the absolute quantitation of GPGV in single putative transmission vectors such us *C*. *vitis* and could be a key factor in epidemiological and plant resistance studies and control strategies.
